# TGFbeta induces apoptosis and EMT in primary mouse hepatocytes independently of *p53*, *p21*^Cip1 ^or *Rb *status

**DOI:** 10.1186/1471-2407-8-191

**Published:** 2008-07-08

**Authors:** Sharon Sheahan, Christopher O Bellamy, Stephen N Harland, David J Harrison, Sandrine Prost

**Affiliations:** 1 Division of Pathology, Queen's Medical Research Institute, University of Edinburgh, 47 Little France Crescent, EH16 4TJ, Edinburgh, UK; 2 MRC Centre for Inflammation Research, Queen's Medical Research Institute, University of Edinburgh, Little France Crescent, Edinburgh, UK; 3 Division of Pathology, Edinburgh Cancer Research Centre, Crewe Road South, EH4 2XR, Edinburgh, UK; 4 Biotransfer Unit, BioSciences Institute, University College, Cork, Ireland

## Abstract

**Background:**

TGFβ has pleiotropic effects that range from regulation of proliferation and apoptosis to morphological changes and epithelial-mesenchymal transition (EMT). Some evidence suggests that these effects may be interconnected. We have recently reported that P53, P21^Cip1 ^and pRB, three critical regulators of the G1/S transition are variably involved in TGFβ-induced cell cycle arrest in hepatocytes. As these proteins are also involved in the regulation of apoptosis in many circumstances, we investigated their contribution to other relevant TGFβ-induced effects, namely apoptosis and EMT, and examined how the various processes were interrelated.

**Methods:**

Primary mouse hepatocytes deficient in *p53, p21 *and/or *Rb*, singly or in combination were treated with TGFβ for 24 to 96 hours. Apoptosis was quantified according to morphology and by immunostaining for cleaved-capsase 3. Epithelial and mesenchymal marker expression was studied using immunocytochemistry and real time PCR.

**Results:**

We found that TGFβ similarly induced morphological changes regardless of genotype and independently of proliferation index or sensitivity to inhibition of proliferation by TGFβ. Morphological changes were accompanied by decrease in E-cadherin and increased Snail expression but the mesenchymal markers (N-cadherin, SMAα and Vimentin) studied remained unchanged. TGFβ induced high levels of apoptosis in *p53-/-*, *Rb-/-*, *p21*^*cip1*^-/- and control hepatocytes although with slight differences in kinetics. This was unrelated to proliferation or changes in morphology and loss of cell-cell adhesion. However, hepatocytes deficient in both *p53 *and *p21*^*cip1*^were less sensitive to TGFβ-induced apoptosis.

**Conclusion:**

Although *p53*, *p21*^Cip1 ^and *pRb *are well known regulators of both proliferation and apoptosis in response to a multitude of stresses, we conclude that they are critical for TGFβ-driven inhibition of hepatocytes proliferation, but only slightly modulate TGFβ-induced apoptosis. This effect may depend on other parameters such as proliferation and the presence of other regulatory proteins as suggested by the consequences of *p53*, *p21*^Cip1 ^double deficiency. Similarly, *p53*, *p21*^Cip1 ^and *pRB *deficiency had no effect on the morphological changes and loss of cell adhesion which is thought to be critical for metastasis. This indicates that possible association of these genes with metastasis potential would be unlikely to involve TGFβ-induced EMT.

## Background

TGFβ is known to have pleiotropic effects which differ according to cell state and differentiation (reviewed in reference [1]). This includes regulation of proliferation and apoptosis, and stimulation of epithelial-mesenchymal transition (EMT) which together are critical for the development of invasive and metastasis potential.

In the liver TGFβ is released in many settings to act as a critical mediator of responses to injury [2]. It controls the proliferation of hepatocytes [3], induces hepatocytes apoptosis [4] and activates EMT [5-8] which, in certain contexts can actually protect hepatocytes from TGFβ-induced apoptosis [9].

In hepatocellular carcinoma (HCC), there is often production of TGFβ from both malignant hepatocytes and the non-parenchymal cells [10,11]. However, evidence suggests that HCC have acquired a resistance to TGFβ inhibition of proliferation through mechanisms that include decreased TGFβ-receptor II expression [12] or induction of the inhibitory SMAD7 in advanced HCC [12,13]. In HCC of different aetiologies, particularly in livers with chronic viral hepatitis B or hepatitis C infection, P53, P21^Cip1 ^and pRb function are altered [14-21]. We have recently reported that deficiency in these proteins significantly affects hepatocyte responses to TGFβ-induced inhibition of proliferation, and may therefore contribute to resistance to TGFβ [22]. As these proteins are also key regulators of hepatocyte apoptosis [23] and given the association of p53 with metastasis potential [24], we determined whether deficiency in these genes also altered TGFβ effects on morphology, EMT and apoptosis, and investigated the possible relationships between those changes.

## Methods

### Hepatocyte culture

Mouse primary hepatocytes (male, 6–12 weeks old), were isolated by liver perfusion of *Rb*-floxed mice (homozygous for exon 19 of *Rb *flanked by LoxP sequences)[25]*p53-/- *[26] and *p21*^Cip1^-null mice [18] using a standard two-step retrograde procedure [27]. Hepatocytes were purified using Percoll gradient [28] and cultured in supplemented serum-free medium selecting against survival of non-parenchymal cells. [29,30]. Rb-floxed hepatocytes were infected at the time of plating with either a replication-deficient adenovirus expressing Cre-recombinase (Ad-Cre) or with the control adenovirus (Ad-Dl70) using a multiplicity of infection of 10 [23]. This gave rise to isogenic hepatocytes either homozygous for the *floxed-Rb *allele (phenotypically wild-type) or homozygous for deletion of exon 19 of *Rb *(*Rb-null*). Where indicated, hepatocytes were treated daily with 160pM of TGF-β 1 (TGFβ) for the indicated time starting from 24 hours of culture.

All animals used in this study received humane care. The study protocols are in compliance with the UK Home Office regulation and the local institutional policies.

### Quantification of apoptosis

Cells were prepared using Feulgen stain and light green counterstain [31,32]. Briefly, cells were fixed in Boum's fixative, treated with 5M HCl for 45 minutes then stained with Schiff reagent. Apoptosis was quantified according the morphological characteristics of the cells as previously described [33]. Normal hepatocytes are flat, with big pale pink nuclei and green cytoplasm while necrotic hepatocytes have shrunken and distorted nuclei darker pink and no condensation of the chromatin (see additional file 1). Apoptotic cells have condensed, uniformly refractile chromatin with retracted condensed cytoplasm (dark green) often with blebbing. Typically apoptotic bodies are best seen at a different plane of focus (above) normal cells. For each time point of each experiment 500 hepatocytes were counted from 2 independent cultures allowing us to plot a percentage of apoptotic cells +/- SD. Each experiment was repeated at least 3 times with similar results.

Statistical analyses were performed using Minitab 13.0. The proportion of apoptotic cells was arcsine transformed, and differences between means were evaluated with Analysis of Variance (ANOVA). Differences were taken to be significant when p < 0.05. Satisfactory homogeneity of variances was determined with Bartlett's test. Where a significant difference between means was identified with ANOVA, pairwise comparisons were performed using Bonferroni tests for multiple comparisons.

In addition to morphological assessment, activation of apoptosis was confirmed biochemically by quantification of cleaved-caspase 3. Briefly, hepatocytes in culture for 72 and 96 hours, treated or not with TGFβ, were fixed in acetone/methanol. Cleaved-caspase 3 was labelled using a rabbit polyclonal antibody (9661S, New England Biolabs, UK) (1/100 overnight) and red-fluorescent secondary antibody (Alexa555 goat antirabbit, molecular probes, UK).

### Immunohistochemistry

Control (wt) and *Rb *-/- hepatocytes in culture for 24 hours on Permanox 2-well chamberslides (LabTech, UK) were treated daily or not with TGFβ(160pM of TGF-β1). 72 hours after treatment (96 hours after plating) the hepatocytes were fixed in Acetone/Methanol (50/50 V/V). After Avidin/Biotin block (Vector, UK), the slides were incubated with a rabbit polyclonal anti E-cadherin antibody (clone NCH38, DAKO UK), rabbit monoclonal anti CK18 (clone E431-1, AbCam, UK), mouse monoclonal anti-SMAα (clone 1A4, Sigma UK) or rabbit polyclonal anti N-cadherin (AbCam, UK), followed by the appropriate biotinylated secondary antibody (Dako, UK) and revealed using ready-to-use peroxidase stain (Vector, UK) and counterstained with haematoxylin.

### Real time RT-PCR

RNA was extracted using Qiagen RNeasy mini kit according to manufacturers' instructions. The concentration and purity of RNA was determined using a NanoDrop^® ^ND-1000 spectrophotometer (NanoDrop Technologies DE, USA). Total RNA (up to 1 μg) was reverse transcribed using Superscript II reverse transcriptase (Invitrogen, UK) in a reaction mix consisting of: 4 μl of 5× buffer, 2 μl DTT (0.1 M), 0.5 μl dNTPs (10 mM), 2 μg of random hexamers, 0.5 μl (100 U) Superscript II and 0.5 μl (20 U) RNAse inhibitor (all Invitrogen, UK). The resulting cDNA was diluted 1:100 ready for PCR.

Expression of both *E-cadherin *and *Snail *was determined using SYBR green master mix (Invitrogen, UK) and the following primers: E-Cadherin: Forward primer ACT GTG AAG GGA CGG TCA AC; Reverse primer GGA GCA GCA GGA TCA GAA TC. Snail: Forward primer CCA CTG CAA CCG TGC TTT T; Reverse primer TCT TCA CAT CCG AGT GGG TTT. Pre-designed 18S primer/probe (Applied Biosystems, UK) was run as an internal control using a TaqMan master mix (Invitrogen, UK). Each system was run under standard conditions on an ABI 7500 detection system

## Results

### TGFβ induces a high level of apoptosis in hepatocytes deficient in p53, p21 and/or Rb, but the sensitivity is genotype-dependent

TGFβ can induce apoptosis in certain cell-types, including hepatocytes [4,34,35]. Given the role of P53 and pRb, respectively as a positive and a negative regulator of apoptosis induced by a variety of stimuli [36,37], we investigated whether the absence of either gene alters the susceptibility of hepatocytes to TGFβ-induced apoptosis.

**Figure 1:**
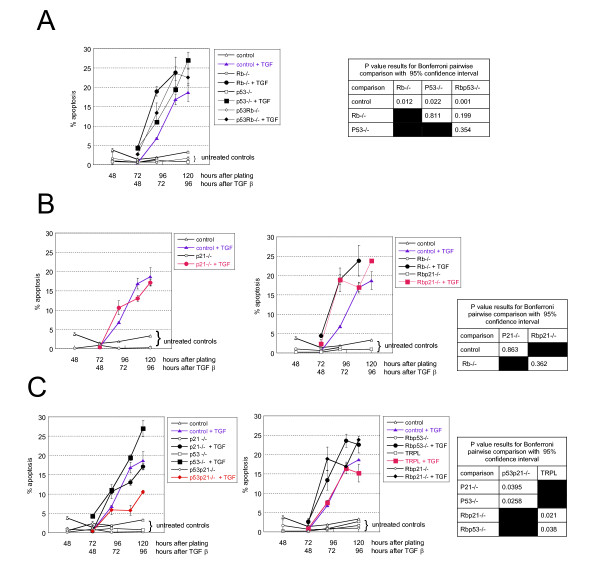
**Effect of *p53*, *p21*^Cip1 ^and *Rb *deficiency on hepatocyte sensitivity to TGFβ-induced apoptosis**. Where appropriate cells were treated with TGFβ from 24 hours in culture. For apoptosis curves in A, B & C, each time point is the average percentage of apoptotic cells +/- SDV obtained from two cultures where 500 cells were counted. The experiment was repeated 3 times with similar results. Control cells are shown in all graphs; in blue control cells treated with TGFβ. Untreated controls are shown with open symbols. The tables show the p values of statistical analysis for pairwise comparison of apoptosis in the genotype of interest (see text and methods). **A**: *p53 *&*Rb *deficiency increase sensitivity to apoptosis. The curves show the % of apoptotic cells in control (▲,△); *p53-/- *(□,■); *Rb-/- *(○,●); and *p53-/- Rb-/-*(◇, ◆) cells treated (plain) or not (open) with TGFβ. **B**: *p21*^Cip1^-deficiency has no effect on *Rb *-/- cells. The curve shows the percentage of apoptosis in cells of indicated genotypes either wild type (black/blue) or deleted for *p21*^Cip1 ^(red) treated or not with TGFβ. **C**: Cells deficient in both *p53 *and *p21*^Cip1 ^are less sensitive to TGFβ-induced apoptosis. Compare black symbols (single nulls for *p53 *or *p21*^Cip1^) with red symbols (deficient in both *p53 *and *p21*^Cip1^).

We found that hepatocytes of all tested genotypes undergo apoptosis after treatment with TGFβ. Interestingly, apoptotic cells were observed earlier in *p53-/-, Rb-/- *and double null cultures (p53Rb-/-) compared with wild-type (4.41; 4.26 and 2.71% versus 0.52% in untreated wild type control 48 hours after treatment) (figure 1A) indicating that the loss of *Rb *or *p53 *sensitises hepatocytes to TGFβ-induced death.

**Figure 2:**
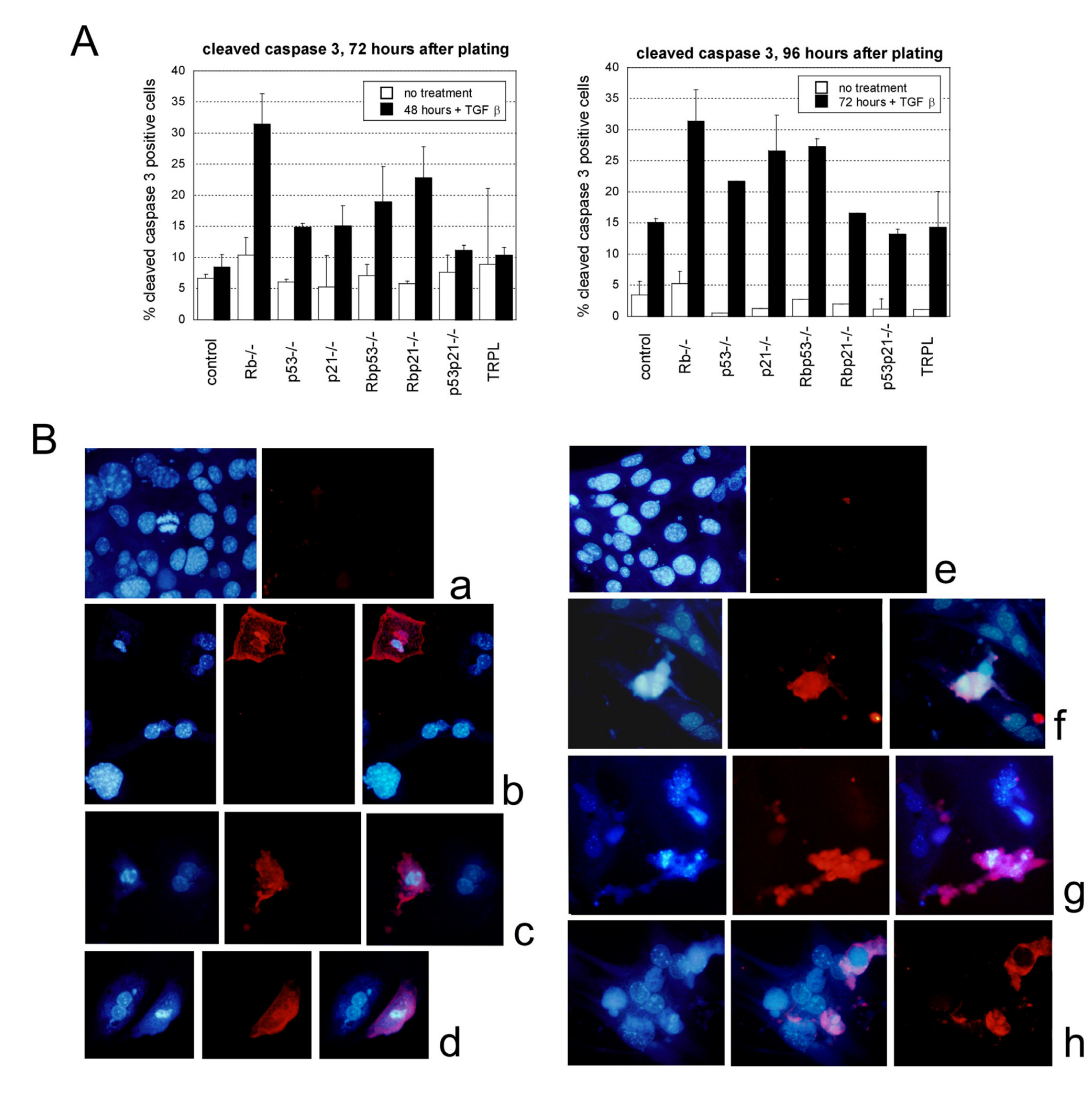
**Caspase 3 cleavage in hepatocytes of different genotypes**. Cleaved-caspase 3 was labelled using red immunofluorescence and the proportion of positive cells quantified by counting 3 × 20 fields of view per genotype. B: The figure shows representative photos of (a, b) untreated hepatocytes 72 hours after plating (here *Rb-/-*); (c, d) hepatocytes in culture for 72 hours treated with TGFβ for 48 hours (p *53-/- *& control); (e) untreated hepatocytes 96 hours after plating (*p21*^*cip1*^-/-); (f, g, h) hepatocytes in culture for 96 hours treated with TGFβ for 72 hours (*p53 p21*^*cip1*^*-/*-, control &*p21*^*cip1*^-/-).

We then studied the effect of *p21*^Cip1 ^deficiency (figure 1B). The absence of functional P21^Cip1 ^had no effect on TGFβ-induced apoptosis in hepatocytes with functional P53 (control and *Rb*-/-) (figure 1B). By contrast, regardless of *Rb *status, deficiency in both P53 and P21^Cip1 ^decreased the sensitivity to TGFβ (decreased apoptosis both in double *p53-/-p21*^Cip1^-/- and triple-nulls *p53-/-Rb-/-p21*^Cip1^-/-) (figure 1C). These differences in apoptosis levels were confirmed by labelling the cells with an antibody specific for cleaved caspase 3 (ASP175) (figure 2). The results were concordant with the morphology data: after 48 hours of treatment with TGFβ (72 hours in culture) control, *p53 p21*^*cip1*^*-/- *and TRPL hepatocytes had similar low levels of cleaved-caspase 3 (figure 2A). Hepatocytes of all other genotypes exhibited higher levels of cleaved-caspase 3, with the highest level observed in *Rb-/- *cells. This is in accordance with the kinetics of appearance of cells with apoptotic morphology (figure 1). After 72 hours of treatment (96 hours in culture) the levels of cleaved-capsase 3 increased in hepatocytes of all genotypes but control, *p53p21-/- *and TRPL showed the lowest levels of cleaved-caspase 3 positivity.

We have previously reported that *p53*, *p21*^Cip1 ^and *Rb *deficiencies lead to increased proliferation albeit of different magnitude depending on the genotype [23]. More recently, we have shown that these genes are also involved in the regulation of TGFβ-induced cell cycle arrest, but again to various degrees [22]. It has been suggested that increased cell cycle activity can lower the apoptotic threshold which would explain the early onset of apoptosis in *p53-/- *and *Rb-/-*. The current observation that apoptosis starts when the first untreated cells enter mitosis (90 to 96 hours after plating in wild-type, and around 72 hours in *p53-/- *and *Rb-/- *cells) might indicate that TGFβ induces a coordinated regulation of growth and apoptosis. However the level of proliferation and apoptosis were found to be unrelated in these cells (additional file 2A). In fact, regardless of *p53 *and *Rb *genotypes, hepatocytes deficient in *p21*^Cip1 ^always proliferate more but showed less apoptosis after TGFβ-treatment than their *"wild type for p21*^Cip1^*"*counterpart (additional file 2B follow blue arrows). By contrast deletion of *Rb *always increased proliferation and apoptosis after TGFβ, regardless of the other genes' status (additional file 2B, follow purple arrows).

### TGFβ induces morphological changes regardless of genotype

**Figure 3:**
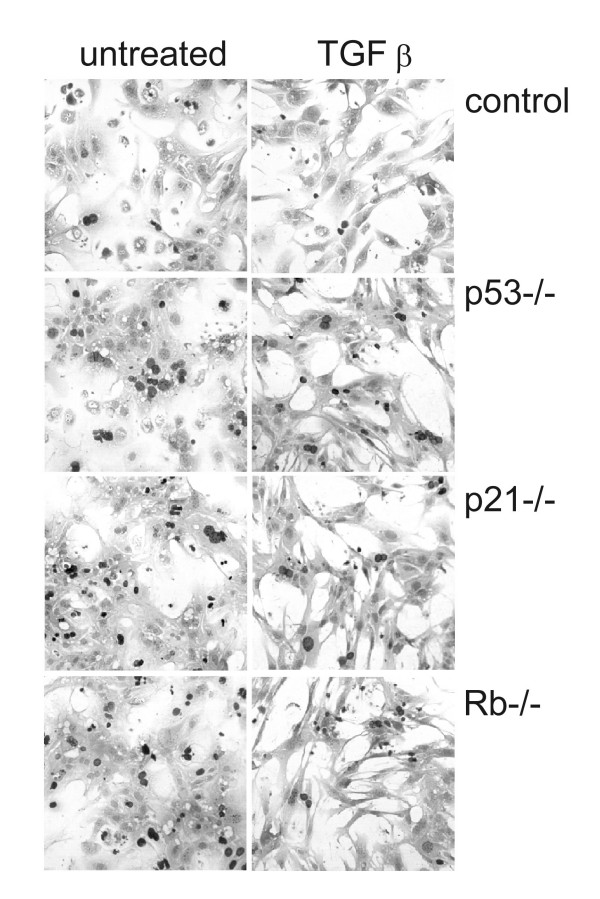
**TGFβ induces morphological changes regardless of genotype**. The figure shows representative photos of control, *Rb*-/-, *p53*-/- and *p21*^Cip1^-/- cells 72 hours after plating, TGFβ-treated or not for 48 hours. The cells were cultured in the presence of BrdU to assess proliferation indexes for another study [23]. The dark nuclei represent immunopositivity for BrdU. Negative nuclei are paler.

TGFβ stimulates production of extracellular matrix (ECM) proteins and their integrin receptors [38], thus affecting cytoskeletal structure, cell shape, and cell spreading, which in turn regulate gene expression and cell growth [39,40]. We observed that TGFβ-treated hepatocytes exhibited dramatic changes in cell shape and spreading. Untreated hepatocytes cultured on plastic coated with fibronectin spread to form a monolayer of cobblestone epithelioid cells. By contrast, TGFβ-treated cells became elongated with reduced cytoplasm and assumed a fibroblast-like morphology (figure 3). These changes occurred regardless of genotype and are similar to those described as epithelial to mesenchymal transition (EMT). We therefore investigated the expression of various proteins that characterise EMT [41].

### TGFβ does not induce complete EMT

**Figure 4:**
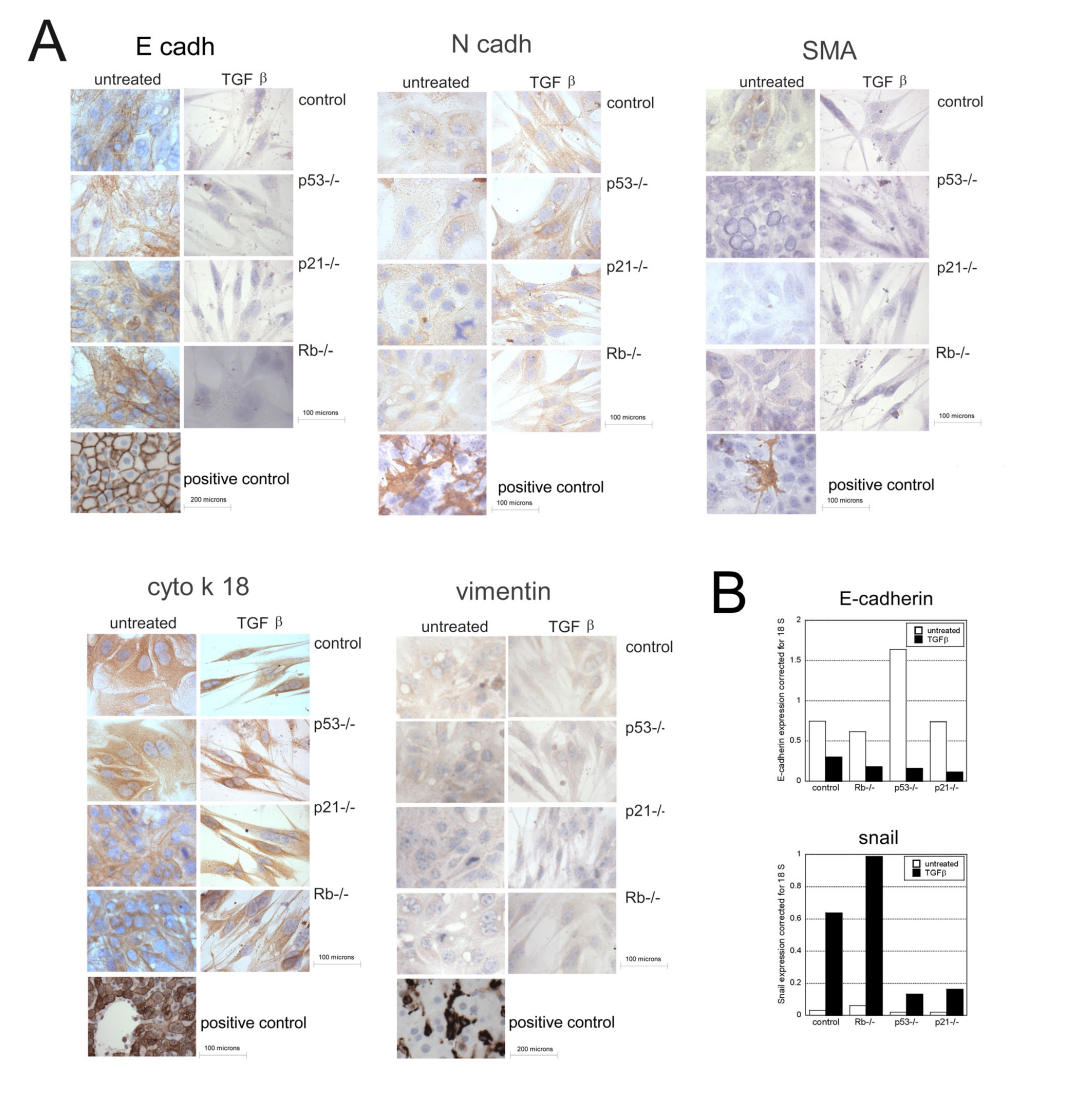
**TGFβ decreases E-cadherin expression**. A: The figure shows representative photos of control, *Rb*-/-, *p53*-/- and *p21*^Cip1^-/- cells 96 hours after plating, TGFβ-treated or not for 72 hours labelled for E-cadherin, N-cadherin, SMAα, vimentin and CK18. Positive controls: *E-cadherin*: membrane staining of hepatocytes; *N-cadherin: *mouse primary hepatic stellate cells; SMAα: mouse primary hepatic stellate cell; *cytokeratin 18*: staining of hepatocytes but not sinusoidal cells; *vimentin*: intense staining of sinusoidal cells but not hepatocytes. B: *E-cadherin *and *snail *expression corrected for 18S amplification in hepatocytes treated or not with TGFβ for 72 hours. Repeat real time RT-PCR with independent samples showed similar results except for *snail *expression in *p21*-/- which was already high and did not further increase after TGFβ-treatment.

EMT is characterised by changes in expression of various proteins, in which downregulation of E-cadherin and concomitant expression of N-cadherin represents defining events ([42] and therein). We analysed the expression of these proteins, together with the expression of vimentin and alpha-smooth muscle actin (SMAα), also expected to increase during EMT (figure 4A) and cytokeratin 18 (CK18), an epitheial hepatocyte marker. As expected, primary hepatocytes expressed E-cadherin and CK18 but not vimentin, N-cadherin or SMAα. After TGFβ-treatment, E-cadherin staining was negative in all genotypes. By contrast there were no changes in expression of N-cadherin, SMAα and vimentin (all negative) or cytokeratin 18 (positive). To confirm these data, we studied *E-cadherin*, *Snail *(a key regulator of E-cadherin expression during EMT) and *N-cadherin *gene expression. Using real time PCR we found that *E-cadherin *expression decreased with TGFβ-treatment while S*nail *expression increased in cells of all genotypes (figure 4B). *N-cadherin *expression was barely detectable and no increase in expression was observed after TGFβ-treatment (data not shown).

## Discussion

TGFβ has pleiotropic effects ranging from regulation of proliferation and apoptosis to morphological changes and mesenchymal transition (EMT). Our evidence suggests that these effects may be interconnected: for example, TGFβ stimulates the production of extracellular matrix proteins and their receptors [38], affecting cytoskeletal structure, cell shape, and cell spreading, which in turn regulates gene expression and cell proliferation [39,43].

We have recently reported that P53, P21^Cip1 ^and pRB, three critical regulators of the G1/S transition are variably involved in TGFβ-induced cell cycle arrest in hepatocytes [22]. As these proteins are also involved in the regulation of apoptosis in many circumstances [36,37], we investigated their contribution to other TGFβ-induced effects, namely apoptosis and EMT, and examined how these different processes were interrelated.

One of TGFβ properties is to induce morphological changes associated with epithelial to mesenchymal transition (EMT), which is characterised by downregulation of epithelial markers concomitant with upregulation of mesenchymal markers [44-46]. Mouse primary hepatocytes of all genotypes expressed high levels of E-cadherin and CK18, two epithelial markers. After treatment with TGFβ, the morphological changes observed were consistent with that seen during EMT: E-cadherin expression decreased sharply while *snail *expression increased. However, mesenchymal markers expression was not significantly increased (N-cadherin, vimentin, SMAα) and CK18 remained unchanged. Therefore the morphological changes were only associated with changes in E-cadherin expression, and this is most probably driven by *snail *[47] and reflect loss of epithelial differentiation rather than mesenchymal transition. Why mesenchymal markers were not expressed is unclear, but longer exposure (4 to 6 days) to several signals may be required for completion of EMT [48]. In any case, this occurred for all genotypes suggesting that P53, P21^Cip1 ^and pRB are not involved in these changes. Nevertheless, morphological changes and proliferation have been shown to be correlated. For example, in rat hepatocytes, morphological changes induced by TGFβ-treatment govern proliferation through alteration of p27^KIP1 ^and p21^Cip1 ^expression [49]. In our cells, TGFβ-treatment did not upregulate p27^KIP1 ^[22]. The morphological alterations also occurred similarly without *p21*^Cip1^, suggesting that these cyclin-dependent kinases (CDKI) are not required for this TGFβ-effect in primary adult mouse hepatocytes. Furthermore, the morphological changes occurred irrespective of genotypes which we have previously reported have differing proliferation index [23] and responses to inhibition of proliferation by TGFβ [22]. This demonstrates that TGFβ-signalling to morphological changes is different to its growth inhibitory signalling in primary mouse hepatocytes. A similar observation has recently been reported for human keratinocytes [50].

TGFβ is well described as a trigger for apoptosis in hepatocytes [35] and high level of apoptosis was observed regardless of genotype studied. Apoptosis appeared somewhat later than in other published studies where high levels are usually reported after 48 hours of TGFβ; the fact that primary hepatocytes in our system are almost synchronous in terms of entry from quiescence to cell cycle on plating [22,23,32] may be a factor and supports the hypothesised influence of cell cycle stage on TGFβ-induced apoptosis. In our study, apoptosis triggered by TGFβ occurred earlier in *p53 *and *Rb*-deficient cells than in control cells, showing an increased sensitivity. It has been suggested that increased cell cycle activity may lower the threshold for apoptosis. More specifically, the abundance of pRb and its level of phosphorylation have been shown to correlate with the threshold for TGFβ-induced apoptosis in hepatocytes [51]. In that study, Fan *et al *reported an enrichment of the G2/M population after TGFβ-treatment and proposed that TGFβ-induced apoptosis occurred in G1 or S phase [51]. In our system, using near synchronous adult primary hepatocytes, TGFβ-treatment triggers a strong and sustained G1-arrest in control cells [22] (less than 1% cells entering S phase) supporting the hypothesis that TGFβ-induced apoptosis occurs in G1. However, the earlier onset of apoptosis in *p53 *and *Rb-null *cells correlates with the peak of S phase and the appearance of the first mitosis suggesting that in cells escaping TGFβ-induced cell cycle arrest, another exit to apoptosis may exist in S phase and G2/M. Although this could contribute to the higher level of apoptosis in *Rb-/- *and *p53-/- *hepatocytes [23] it is clearly not the case in *p21-/- *hepatocytes: we have previously reported that *p21*^*cip1 *^-/- hepatocytes proliferate more and enter S phase earlier than *p53 *or *Rb-/- *cells [23] and that a substantial proportion of *p21*^*cip1*^-/- cells treated with TGFβ are able to enter S phase (10.3% +/- 3.3 in *p21-/- *versus 0.70% +/- 0.42 in control hepatocytes; ([22] and additional file 2). Despite this sustained proliferation in *p21*^*cip1*^-/- cells, apoptosis was of similar level to that of control cells. Similarly, *p21*^*cip1*^*Rb-/- *cells proliferate more than *Rb-/- *cells after TGFβ-treatment (21.1% +/- 0.26 versus 9.05% +/- 3.17 respectively, additional file 2), yet apoptosis was not significantly different in those cells. This may suggest that the presence of *p21*^*cip1*^, and more particularly expression of cytoplasmic P21^Cip1^after TGFβ-treatment [22] which has been associated with apoptosis [52], sensitises hepatocytes to TGFβ-induced apoptosis independently to the proliferation index.

In fact, comparison of all genotypes revealed no simple relationship between the level of proliferation and the amount of apoptosis (additional file 2). This therefore suggests that other parameters are important and that higher proliferation itself can not predict cells' sensitivity to TGFβ.

Interestingly, apoptosis induced by TGFβ in *p53*-null cells was at least partially, dependent on P21^Cip1 ^(regardless of *Rb *genotype) as double deficiency in *p53 *and *p21*^*cip1 *^decreases the level of apoptosis. This effect is specific to TGFβ as it is not observed when apoptosis is induced by other means such as UV-induced DNA-damage (data not shown).

Various recent reports have shown an association between resistance to apoptosis and EMT in hepatocytes [8,9,53]. In one study, TGFβ treatment of a subpopulation of fetal rat hepatocytes resulted in EMT which was associated with resistance to apoptosis. By contrast, adult rat hepatocytes which did not undergo EMT, died by apoptosis [54]. In our system, the morphological changes and loss of cell adhesion may be insufficient to provide resistance, or the pathway may be different in adult primary mouse hepatocytes. This is suggested by the recent study from Ju et al. [55] that showed that Smad2 deficiency leads to EMT but does not affect apoptosis in adult primary hepatocytes.

## Conclusion

During carcinogenesis, growth inhibitory responses to TGFβ are often lost, and insufficient apoptosis has been associated with the development of hepatocellular carcinoma [56]. P53, P21^Cip1 ^and pRb are well known regulators of both proliferation and apoptosis in response to a multitude of stresses. In a previous study, we found that *p53*, *p21*^*cip1*^and *Rb *deficiencies decreased the sensitivity of primary hepatocytes to TGFβ-driven cell cycle arrest. *Rb *deficiency had the strongest effect which was independent of the presence of *p53 *and *p21*^*cip1 *^[22]. Our present results reveal a more subtle involvement of theses genes in the regulation of TGFβ-induced apoptosis. Although differences in the onset of apoptosis were observed in the different genotypes, all but *p53 p21*^*cip1 *^*double null *remained very sensitive to apoptosis induced by TGFβ. We therefore propose that *p53 *and *Rb *have only slight modulatory effect on TGFβ-induced apoptosis and that other parameters including proliferation index, presence of P21^CIP1 ^and double deficiency in *p53 *and *p21*^*cip1 *^are likely to be more important.

EMT is thought to be critical for metastasis. Increased *snail *expression and decreased E-cadherin, associated with changes of morphology occurred irrespective of *p53*, *p21*^Cip1 ^and *pRb *genotypes without mesenchymal transition, suggesting that the association of *p53 *deficiency with metastasis in the liver [24] for example does not correlate with a change in the ability of hepatocytes to undergo loss of cell adhesion induced by TGFβ.

## Abbreviations

Epithelial-Mesenchymal transition: EMT; cytokeratin 18: CK18; Hepatocellular carcinoma: HCC; Standard deviation: SD; Alpha-smooth muscle actin: SMAα; Transforming growth factor β: TGFβ; 5-bromo-2-deoxyuridine: BrdU; *p53 p21*^*cip1 *^*Rb triple null *genotype:TRPL.

## Competing interests

The authors declare that they have no competing interests.

## Authors' contributions

SS initiated the study and carried out some of the experiments, COB contributed to the experimental design, interpretation of data, supervision and gave critical review of the manuscript, SNH carried out some of the experiments, DJH contributed to the experimental design and gave general supervision and funding support, SP made substantial contribution to the conception & design, acquisition and interpretation of data and wrote the manuscript.

## Pre-publication history

The pre-publication history for this paper can be accessed here:



## Supplementary Material

Additional file 1 **Representative photos of primary hepatocytes necrosis and apoptosis**. Our method of quantifying apoptosis and necrosis is based on the morphological characteristics of the cells. Those have been well described, by Kerr JF, Wyllie AH, and Currie AR who first described apoptosis in 1972 (Apoptosis: a basic biological phenomenon with wide-ranging implications in tissue kinetics.*Br J Cancer *1972; 26: 239–257). Enclosed are photos of normal, apoptotic and necrotic hepatocytes, showing how easy these different cells can be recognised after Feulgen staining and light green counterstain. The colours on the photos are real and have not been modified; the cytoplasm is green and the chromatin pink. Normal hepatocytes are flat, with big pale pink nuclei and green cytoplasm. Necrotic hepatocytes have shrunken and distorted nuclei darker pink and no condensation of the chromatin. Apoptotic cells have condensed, uniformly refractile chromatin with retracted condensed cytoplasm (dark green) often with blebbing. Typically apoptotic bodies are best seen at a different plane of focus (above) normal cells. 1 & 2: photos of the same field showing some apoptotic cells in a different plan of focus compared with normal and necrotic cells. 3: In this photo, showing apoptotic, necrotic and normal hepatocytes, the apoptotic cells in the black rectangles have been taken on a different plan of focus to show the apoptotic bodies. Note that necrotic nuclei is "detaching" from cytoplasm area that is not condensed, whereas apoptotic cells have highly condensed chromatin and cytoplasm. Click here for file

Additional file 2 **Proliferation does not dictate the level of apoptosis**. A: The graph represents the index of proliferation versus the percentage of apoptosis in TGFβ-treated hepatocytes of indicated genotypes. The percentage of proliferation after TGFβ-treatment was calculated by integration of the number of cells incorporating BrDU between 72 and 108 hours after plating (48 and 84 hours of TGFβ) [22]. Using videomicroscopy, we have observed that, in our culture conditions, the apoptotic primary hepatocytes remain attached for many days to the plate. The number of apoptotic cells counted at a given time is therefore a good estimation of the number of cells undergoing apoptosis until that time. The graph therefore shows the % apoptotic cells at 108 hours. A similar analysis was performed with proliferation and apoptosis values for 120 hours with similar results. Note the absence of relationship between proliferation and apoptosis. B: As above with arrows showing the effect of *p53 *(red), *Rb *(purple) or *p21*^Cip1 ^(blue) deficiencies on both apoptosis levels and proliferation for the various genotypes. Click here for file
