# Supplementary material for: TGFbeta induces apoptosis and EMT in primary mouse hepatocytes independently of p53, p21Cip1 or Rb status
Source: BMC Cancer. 2008 Jul 8;8:191. doi: 10.1186/1471-2407-8-191 (PMC2467431; doi:10.1186/1471-2407-8-191)
Supplement: Additional file 1 — Representative photos of primary hepatocytes necrosis and apoptosis. Our method of quantifying apoptosis and necrosis is based on the morphological characteristics of the cells. Those have been well described, by Kerr JF, Wyllie AH, and Currie AR who first described apoptosis in 1972 (Apoptosis: a basic biological phenomenon with wide-ranging implications in tissue kinetics.Br J Cancer 1972; 26: 239–257). Enclosed are photos of normal, apoptotic and necrotic hepatocytes, showing how easy these different cells can be recognised after Feulgen staining and light green counterstain. The colours on the photos are real and have not been modified; the cytoplasm is green and the chromatin pink. Normal hepatocytes are flat, with big pale pink nuclei and green cytoplasm. Necrotic hepatocytes have shrunken and distorted nuclei darker pink and no condensation of the chromatin. Apoptotic cells have condensed, uniformly refractile chromatin with retracted condensed cytoplasm (dark green) often with blebbing. Typically apoptotic bodies are best seen at a different plane of focus (above) normal cells. 1 & 2: photos of the same field showing some apoptotic cells in a different plan of focus compared with normal and necrotic cells. 3: In this photo, showing apoptotic, necrotic and normal hepatocytes, the apoptotic cells in the black rectangles have been taken on a different plan of focus to show the apoptotic bodies. Note that necrotic nuclei is "detaching" from cytoplasm area that is not condensed, whereas apoptotic cells have highly condensed chromatin and cytoplasm. [file 1471-2407-8-191-S1.pdf]

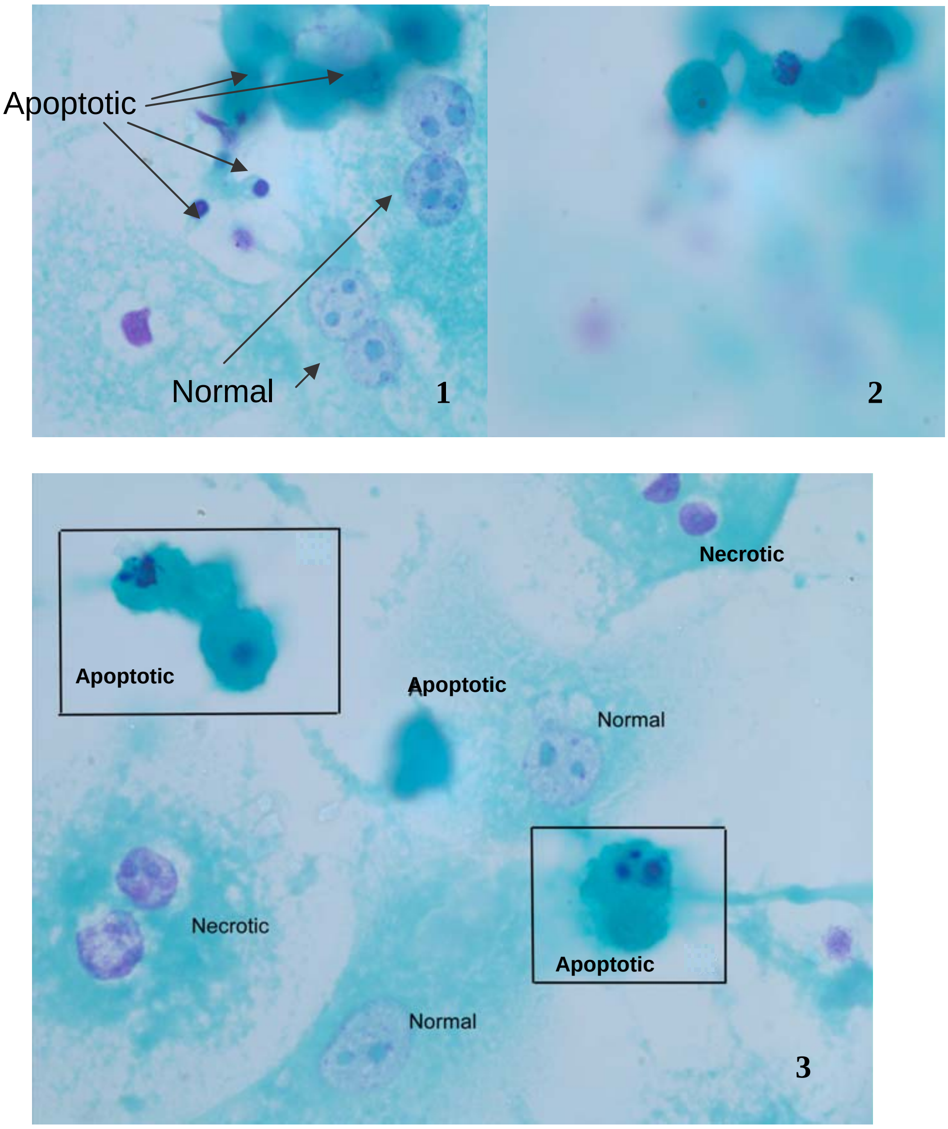

Supplementary figure 1 : **Representative photos of primary hepatocytes necrosis and apoptosis.** Our method of quantifying apoptosis and necrosis is based on the morphological characteristics of the cells. Those have been well described, by Kerr JF, Wyllie AH, and Currie AR who first described apoptosis in 1972 (Apoptosis: a basic biological phenomenon with wide-ranging implications in tissue kinetics. *Br J Cancer* 1972; 26: 239-257). Enclosed are photos of normal, apoptotic and necrotic hepatocytes, showing how easy these different cells can be recognised after Feulgen staining and light green counterstain. The colours on the photos are real and have not been modified; the cytoplasm is green and the chromatin pink.

Normal hepatocytes are flat, with big pale pink nuclei and green cytoplasm.

Necrotic hepatocytes have shrunken and distorted nuclei darker pink and no condensation of the chromatin.

Apoptotic cells have condensed, uniformly refractile chromatin with retracted condensed cytoplasm (dark green) often with blebbing. Typically apoptotic bodies are best seen at a different plane of focus (above) normal cells.

1 & 2 : photos of the same field showing some apoptotic cells in a different plan of focus compared with normal and necrotic cells.

3 : In this photo, showing apoptotic, necrotic and normal hepatocytes, the apoptotic cells in the black rectangles have been taken on a different plan of focus to show the apoptotic bodies.

Note that necrotic nuclei is “detaching” from cytoplasm area that is not condensed, whereas apoptotic cells have highly condensed chromatin and cytoplasm.
