# Supplementary material for: TGFbeta induces apoptosis and EMT in primary mouse hepatocytes independently of p53, p21Cip1 or Rb status
Source: BMC Cancer. 2008 Jul 8;8:191. doi: 10.1186/1471-2407-8-191 (PMC2467431; doi:10.1186/1471-2407-8-191)
Supplement: Additional file 2 — Proliferation does not dictate the level of apoptosis. A: The graph represents the index of proliferation versus the percentage of apoptosis in TGFβ-treated hepatocytes of indicated genotypes. The percentage of proliferation after TGFβ-treatment was calculated by integration of the number of cells incorporating BrDU between 72 and 108 hours after plating (48 and 84 hours of TGFβ) [22]. Using videomicroscopy, we have observed that, in our culture conditions, the apoptotic primary hepatocytes remain attached for many days to the plate. The number of apoptotic cells counted at a given time is therefore a good estimation of the number of cells undergoing apoptosis until that time. The graph therefore shows the % apoptotic cells at 108 hours. A similar analysis was performed with proliferation and apoptosis values for 120 hours with similar results. Note the absence of relationship between proliferation and apoptosis. B: As above with arrows showing the effect of p53 (red), Rb (purple) or p21Cip1 (blue) deficiencies on both apoptosis levels and proliferation for the various genotypes. [file 1471-2407-8-191-S2.pdf]

A

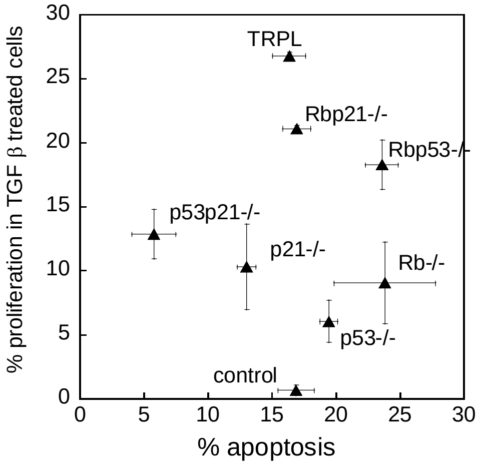

B

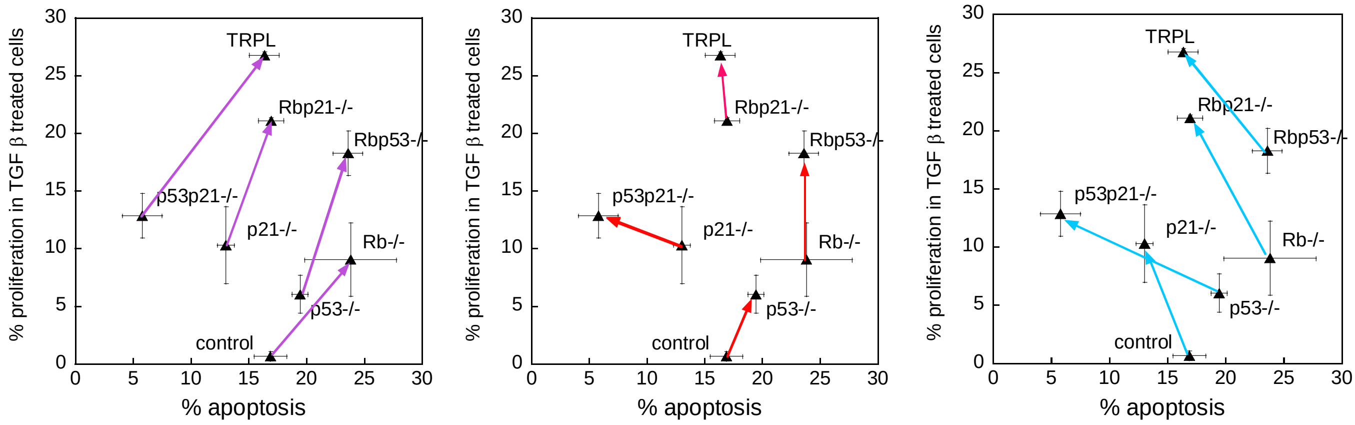

Supplementary figure 2 : **Proliferation does not dictate the level of apoptosis.**

A : The graph represents the index of proliferation versus the percentage of apoptosis in TGFβ-treated hepatocytes of indicated genotypes.

The percentage of proliferation after TGFβ-treatment was calculated by integration of the number of cells incorporating BrDU between 72 and 108 hours after plating (48 and 84 hours of TGFβ) [22] . Using videomicroscopy, we have observed that, in our culture conditions, the apoptotic primary hepatocytes remain attached for many days to the plate. The number of apoptotic cells counted at a given time is therefore a good estimation of the number of cells undergoing apoptosis until that time. The graph therefore shows the % apoptotic cells at 108 hours. A similar analysis was performed with proliferation and apoptosis values for 120 hours with similar results.

Note the absence of relationship between proliferation and apoptosis.

B : As above with arrows showing the effect of *p53* (red) , *Rb* (purple) or *p21*^{Cip1} (blue) deficiencies on both apoptosis levels and proliferation for the various genotypes.
